# Suicidal behaviour in adolescents with affective disorders: A study in a crisis intervention unit (CIU)

**DOI:** 10.1371/journal.pone.0320381

**Published:** 2025-04-01

**Authors:** José Alejandro Valdevila Figueira, María Alejandra Espinoza de los Monteros Andrade, Rocío Valdevila Santiesteban, Andrés Ramírez, Indira Dayana Carvajal Parra, Jimmy Daniel Martin Delgado, Pedro Carlos Martínez-Suárez, Luis Patricio Benenaula Vargas, María Emilia Andrade Hidalgo, Jose A. Rodas

**Affiliations:** 1 Institute of Neurosciences of Guayaquil, Guayaquil, Ecuador; 2 Ecotec University, Guayaquil, Ecuador; 3 Research Network in Psychology and Psychiatry (GIPSI), Guayaquil, Ecuador; 4 Junta de Beneficencia de Guayaquil, Guayaquil, Ecuador; 5 Universidad Católica de Cuenca, Azogues, Ecuador; 6 Universidad Internacional de La Rioja, La Rioja, Spain,; 7 School of Psychology, Universidad Espíritu Santo, Samborondón, Ecuador; 8 School of Psychology, University College Dublin, Dublin, Ireland; Facultad Latinoamericana de Ciencias Sociales Mexico, Mexico

## Abstract

**Background:**

Suicidal behaviour is a critical mental health issue in the adolescent population, often linked to serious emotional problems that leave survivors vulnerable to future risk. Psychological crises in adolescence are primarily associated with relational conflicts, with emotional crises involving depression or anxiety significantly increasing suicidal risk.

**Objective:**

This study aimed to evaluate the rates of suicidal behaviour in adolescents undergoing emotional crises and explore their association with psychiatric diagnoses and demographic factors in Ecuador.

**Methods:**

An observational, correlational study using a quantitative approach was conducted. Data from 252 adolescents admitted to the Crisis Intervention Unit at the Institute of Neurosciences in Guayaquil, Ecuador, between 2011 and 2023 were analysed. Hospitalisation frequencies by year, gender, and associated psychiatric diagnoses were assessed. Data were obtained from each patient’s unified clinical history.

**Results:**

The study found that suicidal behaviour in adolescents hospitalised for emotional crises was most prevalent among females aged 16–18 years. Depressive episodes were the most common psychiatric diagnosis (73%), and cutting was the most frequent method of self-harm, followed by the ingestion of psychotropic substances. A combination of suicidal ideation and attempts was the most frequent presentation (64%), with family conflicts identified as the main source of distress.

**Conclusions:**

The high frequency of suicidal behaviour in adolescent females aged 16-18 years underscores the need for targeted prevention programs addressing emotional crises and stress management in this high-risk group.

## Introduction

### Suicidal behaviour

Suicidal behaviour is a complex, multifaceted, multidimensional, and multicausal phenomenon [1,2]. According to O’Connor et al. [3], suicidal behaviour is defined as a set of behaviours that develop through motivational and volitional phases, including ideation and planning. The integrated motivational-volitional model of suicidal behaviour combines classical models, such as the interpersonal theory of suicide, with more modern theoretical frameworks into a single process that establishes phases and pathways to suicide.

In the pre-motivational phase, predisposing biological factors, life events, and environmental or sociocultural/economic conditions act as drivers. In the motivational phase, suicidal ideation develops, with mediating factors such as hopelessness and feelings of entrapment playing a key role [4]. Finally, volitional moderators such as attitudes towards suicide, access to media, and previous suicide attempts determine the transition from suicidal thoughts to action [4].

Attempted suicide occurs much more frequently than completed suicide, and suicidal ideation is the most common presentation among individuals who die by suicide [5]. The manifestations of suicidal behaviour can vary in intensity, control, duration, lethality, impulsivity, and functionality [6]. Factors such as age, sex, educational level, and region of residence, along with cultural differences, can generate notable variations in its manifestations [5]. Women attempt suicide three times more often than men, but men die by suicide three times more frequently than women, with some exceptions in large, multicultural countries [5].

### Global significance

Suicidal behaviour is one of the leading causes of death among young people aged 15 to 19 worldwide [7], making it essential to understand the factors that lead to attempted suicide in order to develop effective prevention strategies. Previous suicidal behaviour is usually the most common antecedent in individuals who die by suicide, with current estimates ranging between 25% and 50% or 40% and 60%, depending on the country and region [8,9]. A systematic review of European studies found that 16% of cases repeated suicide attempts within a year, with non-fatal outcomes, while only 2% achieved a fatal outcome. Over time, the percentage of deaths by suicide increases by approximately 7% [10]. The United Kingdom presents particular trends in this regard [11].

However, high fatality rates may not always be associated with a history of overt or covert suicidal ideation or previous attempts [12]. This is illustrated by a study of 75 adolescents who had attempted suicide, where 74.7% had no prior history of suicidal behaviour, 18.7% had attempted it once, and 6.7% had made two or more attempts [13,14].

In 2019, suicide was the fourth leading cause of death globally among people aged 15 to 29. More than 1.5 million adolescents and young adults aged 10 to 24 died by suicide, equivalent to over 4,000 deaths per day [15,16]. Data from the World Health Organization (WHO) indicate that almost one million suicides occurred globally in 2019, resulting in more deaths from suicide than from wars, homicides, and chronic non-communicable diseases [17].

Hospitalisation rates for suicide attempts in adolescents remained stable between 2017 and 2019, ranging from 7.9% to 11% [18]. The use of psychotropic drugs is the most common method of attempted suicide, leading to hospitalisation in 67.7% of cases [19]. Adverse social determinants of health are also associated with increased risks of self-harm [20].

A history of previous psychiatric disorders increases the risk of suicidal behaviour [21], as do academic and social pressures, hormonal changes, and genetic factors [22]. Some studies report the presence of prior mental disorders in up to 90% of cases involving individuals who died by suicide, with depression and emotional dysregulation present in 40% of those patients [12,23].

### Ecuadorian context

Half of the completed suicides in Ecuador involve young people under 30 years of age [24]. Suicide currently accounts for 1.7% of annual deaths in the country. Regional data indicate that, over the past twelve months, 4.5% of adolescents attempted to end their lives, 7.5% made a suicide attempt plan, and 14.2% experienced suicidal ideation [24–27]. Between 1990 and 2017, suicide rates among Ecuadorian adolescents were high, with approximately 13,024 deaths during this period, translating into 135,731 years of life lost due to premature mortality [24,25]. Each year, more than a thousand Ecuadorians take their own lives. In 2019, a very high-risk indicator was observed (n women =  322%; n men =  480%). A nationwide longitudinal study found that Ecuador had an average suicide rate of 8.8 per 100,000 inhabitants by 2020, placing it among the ten countries with the highest suicide rates in the region [25,28].

Self-inflicted injuries were the third leading cause of death among Ecuadorians aged 5 to 17 years in 2022 (n =  120; 7.7%) and in the group aged 18 to 29 years (n =  413; 6.8%). In the first group, these injuries were surpassed by land transport accidents (n =  195; 12.5%) and homicides (n =  162; 10.4%), while in the second group, homicides (n =  1933; 32%) and land transport accidents (n =  1308; 21.6%) were more prevalent [29].

In Ecuador, records of suicidal behaviour remain inadequate. From a psychiatric perspective, this phenomenon is often overlooked, as it is not classified as a distinct entity in the DSM-5, which only recognises associated symptoms. Furthermore, the National Institute of Census and Statistics (INEC) from Ecuador uses the ICD-10 system, which identifies suicide and self-harm. However, in cases of completed suicide, only the method used is typically recorded, without further context or contributing factors [30].

### Emotional crises

The high rates of suicidal behaviour among Ecuadorian adolescents underscore the critical role of emotional crises in this population. Emotional crises are temporary yet intense psychological states characterized by overwhelming distress, hopelessness, and emotional instability, often triggered by significant life stressors [1,3,28]. These crises are closely linked to mental health conditions like depression and anxiety, which are key contributors to suicidal ideation and behaviour [8,12,22]. Adolescents experiencing these crises often face heightened impulsivity and difficulty coping with stress, factors that significantly increase their risk of engaging in self-harm or suicide attempts [5,6,21]. In Ecuador, self-inflicted injuries are the third leading cause of death among adolescents aged 5 to 17 years, reflecting the urgent need for targeted prevention and intervention programs [24,29]. The intersection of emotional crises with the broader social and cultural factors influencing adolescent mental health makes this issue a significant public health concern in the country.

The interplay between emotional crises and pre-existing psychiatric disorders, such as depression and anxiety, further exacerbates the risk of suicidal behaviour [12,23]. Emotional crises can amplify feelings of hopelessness and entrapment, mediating the transition from suicidal ideation to action as described in the integrated motivational-volitional model of suicidal behaviour [3,4]. Addressing these crises requires a combination of timely interventions, including crisis counselling, emotion regulation training, and culturally tailored prevention programs, to stabilize adolescents and equip them with effective coping mechanisms. In Ecuador, improving documentation and understanding of suicidal behaviour and its contributing factors—particularly emotional crises—is critical for designing comprehensive, evidence-based interventions that address both immediate risks and long-term psychological well-being [24,27,30]. This approach has the potential to reduce the burden of adolescent suicides and improve outcomes for this vulnerable population.

### The current study

The present study investigates the prevalence and characteristics of suicidal behaviour among adolescents experiencing emotional crises in Ecuador, focusing on those hospitalised at the Crisis Intervention Unit (CIU) of the Neuroscience Institute of Guayaquil between 2011 and 2023. Building on previous research that highlights the multifaceted nature of suicidal behaviour [1,3,4], this study aims to fill a critical gap in understanding the interplay between psychiatric diagnoses, demographic factors, and suicidal ideation or attempts in a high-risk population. By employing a retrospective review of medical records and statistical analyses, the study seeks to clarify the role of emotional crises—intense psychological states often triggered by relational conflicts or life stressors [28]—in contributing to suicidal behaviour. Through this lens, the study aims to provide actionable insights to guide prevention strategies and improve mental health care for adolescents in Ecuador.

## Methods

### Participants

The medical records database from hospitalised patients in the Critical Care Unit (CIU) of the Neuroscience Institute of Guayaquil (INC) between 2011 and 2023 was reviewed. A total of 1026 records were identified, of which 341 cases involved adolescents aged 12 to 18. Hospitalised patients experiencing an emotional crisis involving depression, in whom suicidal ideation or a suicide attempt were present, were included (n =  252). Eighty-nine cases were excluded due to incomplete data in the registries.

### Instruments

On the time of admission, all patients were required to complete an ad hoc demographic questionnaire. The questionnaire collected data on sex (male or female), age, marital status (single, married, or divorced), and educational level (elementary, secondary, high school, or university). It also recorded prior hospitalisations at the INC and the history of previous suicidal behaviour (suicidal ideation or suicide attempt), along with the method used in any attempt. Additionally, participants provided information on their lifetime use of alcohol or drugs, as well as the areas of life they found most concerning (e.g., school, family, legal issues, relationships, or personal matters).

### Procedure

Retrospective data were obtained by analysing the medical records of adolescent patients admitted to the ICU during the study period. The review of clinical records was conducted between 30 August and 5 December 2023. The study protocol was approved by the Human Research Ethics Committee of Luis Vernaza Hospital (CEISH-HLV), with approval code 08-EO-CEISH-HLV-2023. This research adhered to the International Ethical Guidelines for Health-Related Research Involving Humans prepared by the Council of International Organizations of Medical Sciences (CIOMS).

### Statistical analysis

Pearson’s Chi-square test (χ²) was used to compare male and female participants regarding the method of suicide, alcohol/drug use, suicidal behaviour, and main source of emotional crisis. Logistic regression analyses examined the relationship between psychiatric diagnoses (based on ICD-10 classification) and the likelihood of a combination of suicidal ideation and suicidal attempt. This method was chosen to model a dichotomous dependent variable (suicidal ideation or attempt) using independent variables such as psychiatric diagnoses and age.

Diagnoses were compared against depressive episodes, which were chosen as the baseline category due to their high prevalence in the sample (73%) and their well-documented role in suicidal behaviour. This approach allowed us to evaluate whether other psychiatric diagnoses, such as eating disorders or recurrent depressive disorder, were associated with a higher or lower likelihood of suicidal behaviour compared to depressive episodes. Log odds of suicide attempt were reported for each predictor, along with their standard errors (SE), Z statistic values, and corresponding p values, to determine the statistical significance of each predictor. To assess the model fit and calculate its predictive capacity, Cox and Snell’s R² was computed.

## Results

Participants’ diagnoses showed that depressive episodes were the most frequent (n =  185; 73%), followed by bipolar affective disorder (n =  16; 6.3%) and recurrent depressive disorder (n =  24; 9.5%). Other diagnoses included eating disorders (n =  6; 2.4%), dysthymia (n =  1; 0.4%), other mood disorders (n =  13; 5.2%), post-traumatic stress disorder (n =  4; 1.6%), mania with psychotic symptoms (n =  1; 0.4%), obsessive-compulsive disorder (n =  1; 0.4%), and mixed anxiety and depression disorder (n =  1; 0.4%). Only 22 (8.7%) patients reported substance use, including alcohol (n =  1; 0.4%), and other drugs (n =  21; 8.3%).

The methods of suicide varied among participants. Self-inflicted injury was the most common method (n =  81; 32%), followed by ingestion of psychotropic substances (n =  40; 16%), poisoning (n =  9; 3.6%), jumping from a height (n =  6; 2.4%), hanging (n =  5; 2.0%), and the use of firearms (n =  1; 0.4%). Suicidal behaviour was predominantly characterised by a combination of suicidal ideation and attempts (n =  143; 64%), while fewer participants reported only suicidal ideation (n =  79; 35%) or a suicide attempt without ideation (n =  3; 1.3%). For more details, see Table 1.

**Table 1:** Frequency of diagnoses and characteristics of the suicidal behaviour.

	n (%)
ICD-10 Diagnoses	
Depressive episode (F32)	185 (73%)
Bipolar affective disorder (F31)	16 (6.3%)
Food ingestion disorder (F50.8)	6 (2.4%)
Recurrent depressive disorder (F33.3)	24 (9.5%)
Dysthymia (F34.1)	1 (0.4%)
Other mood disorders (F38)	13 (5.2%)
Post-traumatic stress disorder (F43.1)	4 (1.6%)
Mania with psychotic symptoms (F30.2)	1 (0.4%)
Obsessive-compulsive disorder (F42.9)	1 (0.4%)
Mixed anxiety and depression disorder (F41.2)	1 (0.4%)
Substance use	
Drugs	21 (8.3%)
Alcohol	1 (0.4%)
Method	
Hanging	5 (2.0%)
Firearm	1 (0.4%)
Cutting	81 (32%)
Jump into the void	6 (2.4%)
Psychotropic drugs intake	40 (16%)
Poison intake	9 (3.6%)
Suicidal Behaviour	
Suicide Attempt	3 (1.3%)
Suicidal thought	79 (35%)
Suicide attempt and ideation	143 (64%)

**Fig 1:**
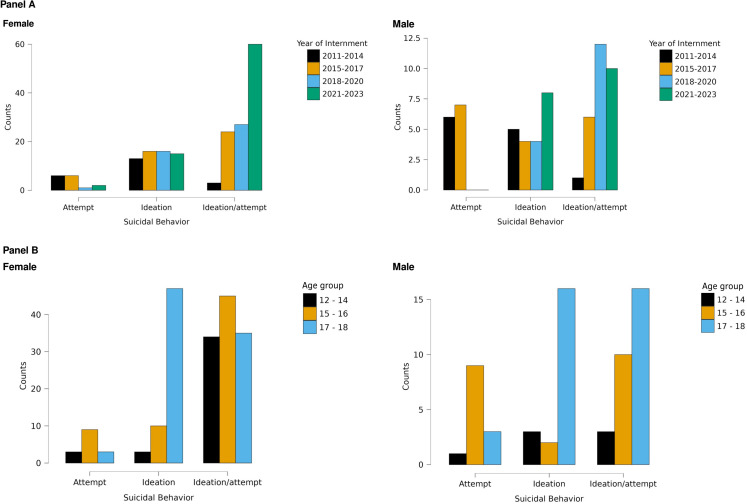
Distribution of Adolescents’ Suicidal Behaviour by Year of Admission and Age. The flow of suicidal behaviour (ideas, attempts, or ideas/attempts) among adolescents, stratified by year of admission (Panel A) and age group (Panel B), divided by gender.

As shown in Fig 1, there is an overall increase in the number of admissions between 2011 and 2023, particularly among women. In a closer inspection, during the 2017–2019 period, the frequency of hospital admissions remained relatively stable, with an average of 24.3 cases per year. However, there is a marked increase in the number of women who thought and attempted suicide between 2021 and 2023.

The number of prior psychiatric hospitalisations ranged from 0 to 5, with a mean of 0.44. Similarly, the mean number of prior suicide attempts was 0.77, with most patients having no history of previous attempts.

The association between gender, drug use, and suicidal behaviour (ideation and attempt) was analysed using Chi-square tests, as detailed in Table 2. The data show that gender was significantly associated with suicidal ideation (p =  0.013) and suicide attempts (p =  0.027). Female adolescents exhibited higher rates of both suicidal ideation and suicide attempts compared to male adolescents. In relation to the suicide method, significant differences were found between male and female participants (p =  0.002). The results did not show a significant association between gender, drug use and the main source of emotional crisis.

**Table 2:** Association between sex and the variables of drug use and suicidal behaviour (ideation and attempt).

Variables	Female (n = 189)	Male (n = 63)	p
**Consumption of drugs/Alcohol**			**0.837**
Drugs	16 (8.5%)	5 (7.9%)	
Alcohol	1 (0.5%)	0 (0.0%)	
**Suicidal behaviour**			
Suicidal ideation	172 (91%)	50 (79.4%)	**0.013**
Suicide attempt	117 (61.9%)	29 (46%)	**0.027**
**Method**			**0.002**
Hanging	1 (0.5%)	4 (6.3%)	
Firearm	0 (0%)	1 (1.6%)	
Cutting	70 (37%)	11 (17.5%)	
Jumping from a height	6 (3.2%)	0 (0.0%)	
Psychopharmaceuticals	29 (15.3%)	11 (17.5%)	
Poison	7 (3.7%)	2 (3.2%)	
**Main source of emotional crisis**			**0.118**
School	1 (0.5%)	2 (3.2%)	
Familiar	117 (61.9%)	33 (52.4%)	
Legal	0 (0%)	1 (1.6%)	
Partner	4 (2.1%)	0 (0.0%)	
Personal	12 (6.3%)	6 (9.5%)	

***Note:*** Pearson’s Chi-squared test (χ^2^)

The Chi-square analyses, summarised in Table 3, reveal several significant associations between demographic variables, methods, and main source of emotional crisis related to suicidal behaviour. While gender was not associated with the type of suicidal behaviour, age groups were, with adolescents aged 17–18 being the most prevalent in both suicidal ideation and attempts. Cutting was identified as the most frequent suicide method, and family conflicts emerged as the most common area of concern associated with suicidal behaviour. Regarding hospitalisation history, 72.6% of adolescents had no prior hospitalisations, 18.7% had been hospitalised once, and 8.7% had two or more prior hospitalisations. Additionally, a greater number of previous hospitalisations was associated with an increased likelihood of engaging in new suicidal behaviours.

**Table 3:** Contributing elements for suicide behaviour.

	Attempt n = 3 (1.3%)	Ideation n = 79 (35%)	Ideation/attempt n = 143 (64%)	p-value^1,2^
**Gender**				**0.40**
Female	3 (100%)	58 (73%)	114 (80%)	
Male	0 (0%)	21 (27%)	29 (20%)	
**Age groups**				**<0.001**
15-16 years	2 (67%)	11 (14%)	55 (38%)	
17-18 years	1 (33%)	62 (78%)	51 (36%)	
12-14 years	0 (0%)	6 (7.6%)	37 (26%)	
**ICD-10 diagnosis**				**0.14**
Depressive episode (F32.2)	3 (100%)	54 (68%)	118 (83%)	
Bipolar affective disorder (F31.4)	0 (0%)	5 (6.3%)	6 (4.2%)	
Food ingestion disorder (F50.8)	0 (0%)	5 (6.3%)	1 (0.7%)	
Recurrent depressive disorder (F33.3)	0 (0%)	11 (14%)	10 (7.0%)	
Dysthymia (F34.1)	0 (0%)	0 (0%)	0 (0%)	
Other mood disorders (F38)	0 (0%)	3 (3.8%)	7 (4.9%)	
Post-traumatic stress disorder (F43.1)	0 (0%)	0 (0%)	1 (0.7%)	
Mania with psychotic symptoms (F30.2)	0 (0%)	0 (0%)	0 (0%)	
Obsessive-compulsive disorder (F42.9)	0 (0%)	0 (0%)	0 (0%)	
Mixed anxiety and depression disorder (F41.2)	0 (0%)	1 (1.3%)	0 (0%)	
**Consumption of drugs/alcohol**				**<0.001**
Drugs	0 (0%)	1 (1.3%)	17 (12%)	
Alcohol	1 (33%)	0 (0%)	0 (0%)	
**Method**				**<0.001**
Hanging	0 (0%)	0 (0%)	5 (3.5%)	
Use of firearm	0 (0%)	1 (1.3%)	0 (0%)	
Cutting	2 (67%)	1 (1.3%)	78 (55%)	
Jumping from a height	0 (0%)	0 (0%)	6 (4.2%)	
Psychotropic drugs intake	1 (33%)	0 (0%)	39 (27%)	
Poison intake	0 (0%)	0 (0%)	9 (6.3%)	
**Main source of emotional crisis**				**<0.001**
School	0 (0%)	0 (0%)	2 (1.4%)	
Familiar	1 (33%)	22 (28%)	107 (75%)	
Legal	0 (0%)	0 (0%)	0 (0%)	
As a couple	2 (67%)	0 (0%)	2 (1.4%)	
Staff	0 (0%)	2 (2.5%)	14 (9.8%)	
**Previous hospitalisations**				**0.002**
0	2 (67%)	72 (91%)	97 (68%)	
1	0 (0%)	4 (5.1%)	34 (24%)	
2 or more	1 (33%)	3 (3.8%)	12 (8.4%)	

^1^ Fisher’s exact test. ^2^ False discovery rate correction for multiple testing.

The logistic regression analyses examined the relationship between various psychiatric diagnoses, according to the ICD-10 classification, and the likelihood of suicidal ideation or attempt. The model began with an intercept of 8.892, which was highly significant (p < .001). This suggested a high probability that suicidal ideation or attempt would occur in the absence of the factors considered. The intercept established the model’s baseline, which was then adjusted by various predictors, such as psychiatric diagnoses and age. According to the results, presented in Table 4, only eating disorder and age presented a significant association.

**Table 4:** Results from the logistic regression analyses.

Predictor		Estimate (B)	SE	Z	p
Intercept		8.892	1.686	52.737	< .001
**ICD-10 diagnosis:**					
	Bipolar affective disorder – Depressive episode	-1.007	0.569	-17.700	0.077
	Eating disorder – Depressive episode	-2.471	1.137	-21.722	0.030
	Recurrent depressive disorder – Depressive episode	-0.611	0.475	-12.870	0.198
	Dysthymia – Depressive episode	-16.242	1.455.398	-0.0112	0.991
	Other mood disorders – Depressive episode	0.291	0.621	0.4684	0.640
	Post-traumatic stress disorder – Depressive episode	-1.807	1.181	-15.294	0.126
	Mania with psychotic symptoms – Depressive episode	-16.242	1.455.398	-0.0112	0.991
	Obsessive-compulsive disorder – Depressive episode	-16.242	1.455.398	-0.0112	0.991
	Mixed anxiety and depression disorder – Depressive episode	-16.242	1.455.398	-0.0112	0.991
**Age**		-0.513	0.102	-50.203	< .001

***Note*:** Logistic regression. Cox and Snell R² =  19%.

## Discussion

Suicidal behaviour has significant and lasting effects throughout the lives of adolescents, yet its impact is often not adequately recognised by the healthcare system of several countries, which is the case of Ecuador. For this reason, the present study aimed to evaluate the rates of suicidal behaviour in adolescents experiencing emotional crises and to examine their relationship with psychiatric diagnoses and demographic factors in the Ecuadorian population.

### Suicide rates

The incidence of suicide among adolescents is increasing globally due to a complex etiopathological interaction between genetic factors and increasingly demanding environmental factors [31–34]. The age of onset of suicidal behaviour appears to be decreasing, likely due to the growing presence of comorbidities [35,36]. In the Americas, high suicide mortality rates have been reported in recent years, with 9,800 deaths between 2015 and 2019, 79% of which were men, who exhibit suicide rates three times higher than women. Suicide is the third leading cause of death among young people aged 20 to 24 years [37]. The age groups 10–19 and 15–23 display distinct patterns of suicide attempts and ideation across the region.

It is important to recognise that the effects of suicidal ideation and attempts extend beyond psychological impact. In Ecuador, the burden of years lived with disability (YLD) due to self-harm is higher among adolescent girls aged 10–14 years (354.93 YLD per 100,000 inhabitants) than among boys. Conversely, among males aged 15–19 years, the impact is significantly greater than among females, reaching 1,109.87 YLD per 100,000 inhabitants [38]. Notable gender differences exist in the impact of suicidal behaviour: while girls attempt suicide more frequently, boys account for the majority of completed suicides.

The differences in impact are multifactorial. Among girls, mental disorders such as personality disorders, bipolar disorder, depressive symptoms, post-traumatic stress disorder (PTSD), and particularly eating disorders (OR 5.27, 95% CI 2.04–13.60) are more prevalent. Additional factors, such as abortions (OR 1.3, 95% CI 1.09–1.55), also play a role. In contrast, boys are more likely to report symptoms related to hopelessness (OR 1.74, 95% CI 1.04–2.94) and disruptiveness (OR 8.78, 95% CI 2.77–27.84), alongside higher rates of alcohol and drug consumption [39]. However, studies suggest that these individual differences are linked to the sociocultural context, including greater exposure to violence and insecurity, socioeconomic inequality, sexism, identity issues, stereotypes, and body image concerns [40].

Cultural factors may play a significant role in shaping the expression and understanding of suicidal behaviour in Ecuador. Cultural norms surrounding family dynamics, societal expectations, and stigma toward mental health issues can exacerbate emotional crises and delay help-seeking behaviours among adolescents. For instance, collectivist values emphasizing family cohesion may increase stress for adolescents experiencing relational conflicts, while stigma associated with mental health conditions could discourage open dialogue and professional intervention. Exploring these cultural dimensions is important for designing prevention strategies that are not only evidence-based but also culturally sensitive, ensuring they are relevant for the target population.

Records from 2001 and 2014 show that Ecuador had an incidence rate of 7.5 and 13.6 per 100,000 for suicide and suicide attempts, respectively, exceeding the rates reported in Colombia (4.7 and 9.4) and Peru (1.1 and 1.9). During that period, suicide accounted for 15–20% of deaths from external causes among these age groups in Ecuador, compared to 11% in Colombia and 7% in Peru [41].

In the present study, women reported higher rates of suicidal ideation and suicide attempts than men, consistent with research suggesting that women are more likely to attempt suicide [24,32,42]. Most hospitalised adolescents (69%) were aged 16–18 years (n =  173), with approximately three women (n =  189; 75%) for every man (n =  63; 25%).

### Factors related to suicide

Age, gender, social and cultural influences are closely linked to suicidal behaviour. However, it is essential to analyse these factors contextually to develop effective preventive and intervention strategies [40]. Mental illness in adolescents often causes shame for both the patient and their family, generating fear of stigmatisation. As a result, symptoms may be hidden or denied, or there may be reluctance to seek help, particularly from non-specialists, which is more common among women [43].

Adolescent depression (especially among girls), conflictual relationships with parents, and academic stress are frequently identified as the leading causes of higher suicide attempt rates among adolescent girls. Adolescents often experience emotional episodes as a chaotic mix of intense emotions, with suicidal behaviour described as a manifestation of profound mental pain and intense anger [44]. Due to their developmental stage, adolescents may lack the coping strategies needed to manage overwhelming irritability, which can lead to verbal or physical aggression towards themselves or others [45].

Impulsivity is another notable feature in emotional regulation, often exacerbated in individuals with suicidal behaviour, influencing their attitudes towards suicidal intent. A study of Ecuadorian adolescents found that impulsivity was a significant predictive factor (β = .200, t =  4.00, p < .001) in the pathways mediating suicidal ideation among adolescents in Guayaquil [46].

Adolescents’ behavioural manifestations, driven by poor impulse control and low frustration tolerance, often escalate into crises that may develop into medical emergencies due to unresolved emotional distress. This study found that most hospitalisations were caused by depressive crises of varying intensities (n =  185; 73.5%). These findings highlight the need to evaluate and treat depressive episodes to prevent suicidal behaviour, confirming the link between depression and suicide observed in this study. Research has also emphasised the prevalence of depression and anxiety disorders as primary contributors to suicide attempts and ideation among adolescents worldwide [12,47,48], reinforcing the need for appropriate interventions to mitigate these risks [14].

In our case, clinical diagnosis did not show a significant association with suicidal behaviour. However, a systematic review identifies mental health disorders as important predictors of suicide risk [40]. A meta-analysis found that a history of mental health issues significantly increases the risk of suicide attempts among young people (OR 3.56), with psychiatric comorbidity identified as a primary risk factor, especially mood disorders (OR 1.54) [49].

Other studies with Ecuadorian adolescents found depression to be the strongest predictor of suicidal ideation (β = .522, t =  11.97, p < .001) [50]. Similarly, Lacomba-Trejo et al. identified depression as both a mediating and predictive factor for suicide in 137 Ecuadorian adolescents; in cases of severe depression, the risk was twice as high (mean =  2.09, SD =  1.93, p < .001) compared to those with anxiety, stress, or low self-esteem [47]. Another study found that 90% of individuals who died by suicide had prior mental health diagnoses. Although no specific disorder predominated, common contributors such as emotional regulation deficits were identified, with depression being associated with up to 40% of the cases [12,23].

The results of this study reveal a pattern of suicidal behaviour characterised by a combination of suicidal ideation and previous attempts, primarily among female participants diagnosed with episodic depression, some of who engaged in drug use and frequently used cutting as a suicide method. Family-related concerns were identified as the most common area of conflict, likely linked to the amount of time spent at home and within the family environment.

Eating disorders were present in only six cases (2.4%). According to the reviewed literature, these disorders are often associated with frequent psychiatric and physical comorbidities [51,52], as well as impairments in physical, social, and occupational functioning [53]. Eating disorders can affect individuals of all ages, ethnicities, and socioeconomic backgrounds [54], although adolescents and young adults are particularly vulnerable, with the average age of onset decreasing [52]. Women are more frequently affected by anorexia nervosa, often developing it at a younger age than other disorders in this group [55,56].

Substance use, however, was significantly associated with suicidal behaviour, with drug use being the most prevalent among adolescents with suicidal ideation and attempts. Strong evidence demonstrates a robust relationship between drug use and an increased risk of suicidal behaviour across all age groups, including adolescence [16]. A study conducted among 1,026 Spanish adolescents aged 14 to 16 years (530 males and 496 females) reported high but variable rates of drug use, with gender differences. Alcohol use was more prevalent among males (11.89%) than females (7.86%) and was associated with tobacco and other drug use. Previous suicide attempts, depressive symptoms, interpersonal problems, and alcohol use were identified as key predictors of suicidal behaviour [41].

The likelihood of suicide increases as alcohol consumption worsens, while abstaining from alcohol is associated with lower levels of suicidal ideation. Furthermore, combined use of drugs and alcohol amplifies suicidal ideation, as reported by studies from Valdevila et al. and Karbeyaz et al. [57,58]. Similarly, substance use and mental disorders linked to substance abuse increase the risk of suicidal behaviour. A regional study by Castaldelli-Maia et al. highlights that Ecuador has one of the highest rates of substance use disorders in the region, particularly related to the consumption of opioids and cocaine. In this context, the prevalence and incidence of opioid use in Ecuador have shown a significant increase between 2010 and 2019, reaching 151 cases per 100,000 inhabitants (95% CI 121–192) [59].

Our sample consisted of adolescents from the four regions of Ecuador: Coast (221), Amazon (3), Galápagos islands (2), and Highlands (26), with the majority coming from the Guayas province (198), likely due to its proximity to the study site. In Ecuador, suicide rates vary by region. In Guayas (within the coastal region), the adjusted suicide incidence is 3.53 per 100,000 inhabitants (3.23–3.99) [27]. A study by Ortiz-Prado et al. [27] found that provinces located in the Highlands region were more affected by suicide than those in the Coast and other lower-altitude regions.

Other studies have also reported a higher prevalence of depression in mountainous regions compared to coastal cities [60–62]. Additionally, a positive relationship between altitude and suicide rates has been suggested, with regions located above 2,500 metres having a higher incidence rate (9.4 per 100,000) than those at lower altitudes (6.3 per 100,000) [63,64]. Unfortunately, no studies have yet explained these variations within Ecuador.

Despite the substantial amount of research on this topic, the relationship between altitude and suicide rates remains speculative [65,66]. It is essential to consider confounding variables in the contextual relationship between altitude and suicide risk, which should be addressed in future local research. These variables include geographic limitations, as well as the social and economic resources available in high-altitude provinces. Other individual factors, such as sociocultural and physical aspects, may also help clarify the geographic variability in depression incidence [67,68].

In our sample, 176 adolescents linked their crisis to conflicts in their environment, with family conflicts being the most common (n =  150; 59.5%), followed by personal conflicts (n =  18; 7.1%) [43]. This suggests that social adaptation issues and family conflicts are associated with a higher likelihood of suicide attempts. Moyano et al.’s study supports these findings by analysing the impact of family ties and support networks on predictive pathways for suicidal ideation in a sample of 395 adolescents from Guayaquil. They found that alienation from the mother (r = .23, p < .001), father (r = .21, p < .001), and peers (r = .21, p < .001) was positively associated with suicidal ideation. Conversely, trust (r = .18, p < .001) and good communication with the mother (r = .10, p < .001) showed an inverse relationship with suicidal ideation [43].

Aspects such as the relationship between adolescents and their mothers are identified as factors that can influence suicidal behaviour among adolescent girls. A mother’s history of suicidal behaviour is a significant risk factor for suicide attempts in female adolescents, suggesting a stronger familial and social influence on girls. This influence may compel adolescent girls to use suicidal methods to express dissatisfaction with social and family expectations, as noted by Collado and Xu et al. [43,69].

A study examining the association between adverse social determinants of health and the risk of self-harm among young people found that 3,262 (1.3%) individuals from a sample of 244,958 youths aged 10 to 17 with a primary psychiatric diagnosis had at least one adverse indicator. These included parent-child conflicts (HR =  1.52, 99% CI =  1.23–1.87) and other family problems (HR =  1.25, 99% CI =  1.01–1.54), both of which were associated with a significantly higher risk of self-harm [20].

### Impact of COVID-19 on suicide rates

An independent analysis of the COVID-19 pandemic period and its impact on adolescent mental health is warranted. This study observed a marked decline in hospital admissions in 2020, consistent with the pattern reported by Lee et al. [70], likely due to lockdown measures that encouraged families to stay home to minimise exposure to the virus. This was followed by a significant increase in cases in 2021 and 2022, aligning with studies showing rising suicide rates during and after the pandemic, attributed to emotional aftereffects and additional stress caused by the situation [43]. The increase in hospitalisations during these post-pandemic years is consistent with reports of rising suicide rates in studies conducted during and after the pandemic period [18,70,71].

During the immediate post-pandemic period, the number of admissions increased considerably compared to 2020. This rise was related to the inability to seek care outside the home without the fear of infection. Carpio-Arias et al. evaluated the impact of mobility restrictions during the pandemic in a study with young people, finding that changes in daily routines, such as reduced movement for work or grocery shopping, were significantly associated with depressive symptoms or eating disorders [72].

The reviewed literature shows that during the COVID-19 pandemic, one in four adolescents experienced depressive symptoms [73]. Additionally, depression during this period was present in approximately 40% of children with specific mental disorders who attempted suicide [12,23]. However, a national suicide study in Ecuador, which included time-series analyses, found that the differences in the number of cases reported by the country’s integrated emergency service (ECU911) were not significantly higher compared to the pre-pandemic period (RR 0.97 [0.92–10.2], * p * =  0.185), with the highest peak occurring after an earthquake in 2016.

Following the onset of the COVID-19 pandemic, the number of hospitalisations decreased (2020; n =  7; 2.8%). Ecuador was one of the countries most affected by COVID-19, with the highest mortality rates in Latin America [24]. During the period of mandatory confinement and self-isolation, a large number of people experienced severe symptoms of depression (20.3%) and anxiety (22.5%), with adolescents reporting the highest rates [74].

Between 2018 and 2019, Ecuador’s Social and Community Support systems reported 7,601 cases of intentional poisoning, 4% (n =  127) of which resulted in death in the general population. A pattern emerged among suicidal adolescents aged 10 to 14 years, with a preference for this method, particularly among girls [47,60].

### Conclusions

Self-injurious behaviours may represent a cry for help [75], often aimed at seeking support [76]. However, these behaviours are usually carried out in secret due to fear of rejection or negative judgement from others [77]. Parents often experience drastic changes in their relationship with their child after the first visible suicidal behaviour or the onset of a mental disorder [78]. The emergence of such behaviours prompts parents to redefine their perception of their child [79], with many struggling to connect with their child’s inner world due to severe communication barriers [80]. Therefore, strategies must be established to enable effective monitoring of adolescents within the family environment, supported by professional guidance.

Self-injurious behaviours among adolescents are a growing global concern, with studies indicating that these behaviours often serve as maladaptive coping mechanisms for emotional distress. While cultural variations influence the way self-harm and suicidal behaviour are perceived and responded to, common risk factors—such as family dysfunction, academic pressure, and social isolation—are widely observed across different countries [8]. In some regions, stigma surrounding mental health and suicide further exacerbates the issue, discouraging adolescents from seeking professional support. The findings of this study, particularly regarding the secrecy of self-harming behaviours and parental struggles in understanding their child’s distress, align with international research that underscores the need for family-centered interventions. Implementing evidence-based strategies, such as parent training programs and psychoeducation initiatives, could bridge the communication gap between adolescents and their caregivers, fostering a more supportive environment for mental health recovery. However, fundamental issues related to Ecuador’s demographics, such as the presence of extreme poverty and violence, high prevalence of drug abuse or low education levels [81] prevent such strategies from being adequately implemented.

This study provides an in-depth exploration of suicidality among adolescents in Ecuador, addressing a relatively understudied area. By examining variables such as age, sex, self-harm methods, education level, marital status, discharge diagnoses, and substance use, it sheds light on the complexities underlying adolescent suicidality. The study underscores the importance of the crisis intervention unit within Ecuador’s mental health framework [69]. It highlights the relationships between gender, age, suicidal ideation, and attempts, as well as their connection to depressive episodes, stressing the need for early detection and treatment in adolescents. The findings also reveal a post-pandemic surge in suicidal behaviour, emphasising the importance of mental health support during crises. Additionally, the study identifies a strong association between drug use, suicidal ideation, and attempts, reinforcing the need for substance abuse prevention and intervention programs. This research contributes to filling the knowledge gap on adolescent suicidal behaviour in Ecuador and offers a foundation for developing targeted interventions and policies to address this significant public health issue.

In conclusion, adolescents hospitalised in crisis situations with suicidal behaviour are often affected by episodic depression accompanied by suicidal ideation and self-injurious attempts. Suicidal behaviours are more prevalent among female adolescents from conflict-ridden family environments, with self-harm and psychotropic drug intake being the most frequent methods. Future research should focus on working with this high-risk group through programs that promote emotional education and the development of psychological resources for coping with stress. Additionally, efforts should be directed towards improving early detection of affective disorders and refining suicide risk prediction models to better serve vulnerable populations.

### Limitations of the study

This study presents several limitations that should be considered when interpreting the findings. First, the low number of participants in some predictors included in the logistic regression model, such as specific diagnoses and alcohol or drug use, limits the robustness of the analyses and the generalizability of the results. For instance, in several categories, the number of participants was as low as one, which may have reduced the statistical power of the regression model and affected the reliability of the findings.

Second, the lack of a standardized protocol for data collection across the study period posed challenges to ensuring consistent and comprehensive data. This limitation particularly affected the inclusion of potentially relevant variables, such as detailed family or personal medical histories, which could provide deeper insights into the factors contributing to suicidal behaviour. Additionally, the presence of parents during interviews may have restricted adolescents from openly disclosing sensitive information, such as alcohol and drug use or previous emotional issues, leading to possible underreporting of these variables as highlighted in previous studies [82].

Future research should address these limitations by employing standardized and comprehensive data collection protocols and ensuring larger sample sizes for underrepresented predictor categories. These improvements will help enhance the robustness and generalizability of future studies on adolescent suicidal behaviour.
